# Used-car market dataset for Latvia 2018

**DOI:** 10.1016/j.dib.2018.12.075

**Published:** 2018-12-28

**Authors:** Valerijs Skribans

**Affiliations:** Riga Technical University, Latvia

## Abstract

Used-car market monitoring was started in 2018, 03 January, when Federal Administrative Court in Leipzig (Germany) allowed cities and communes in Germany to impose bans for diesel cars in order to reduce the level of nitric oxide in the air. This decision can radically change used-car market in the EU, because Germany massive exports used cars to Eastern Europe. The database shows the influence of the Germany Court decision to the used-car market in Latvia, as part of the EU market. The database represents the used-car market monitoring in Latvia during 2018. In Latvia 20–22th. used-car are offered for sale each month. The database reflects the observation dynamics of 12 months. In the observations there are included main used-car characteristics such as price, first registration date, motor cubic capacity, motor type, gearbox type, mileage, color, producer (brand), model, body types, (following) technical inspection data, set (features) of cars and so on.

**Specifications table**

**Table:** 

Subject area	*Economics, Econometrics and Finance*
More specific subject area	*Business and management, marketing, market study, econometrics*
Type of data	*Table*
How data was acquired	*Market advertisements in internet monitoring with Data Mining technologies, for data collection used Rcrawler library*
Data format	*Raw*
Experimental factors	*Data was groped by months according collection process, no changes were made to the data*
Experimental features	*Latvian used-car market not so big, so it is possible to observe market advertisements in internet with Data Mining technologies. There was collected average 21 thousands offers per months of used-car in Latvia in 2018 for 12 month.*
Data source location	*Latvia, European Union*
Data accessibility	*Data is attached with this article*
Related research article	*Skribans V., Hegerty, S.W. Data mining application for used-car market analysis (In press)*[1]

**Value of the data**

• The data reflects the used-car market and preferences of cars buyers in Latvia.
• It is possible to analyze the effects of diesel-gate and German legal restrictions on the industry in Latvia, based on it, it is possible draw conclusions about the long-term sustainable development of the car manufacturing industry in EU (because Germany massive exports used cars to Eastern Europe, including Latvia). The data set confirms that in situation, when in Germany ratio of diesel cars decrease, in Latvia the ratio increase for used-car only. Based on data it possible forecast, when last diesel car in Latvia (and, probably, in Europa) will be utilized.
• Data can be used for illegal economy estimation in Latvia on used-car market (for it, it is needed to compare data with official institution data [2]).

## 1 Data

Database consists of Latvian used-car market advertisements monitoring, total 258.6 th. observations. The database fields are cars price, first registration date, motor cubic capacity, motor type, gearbox type, mileage, color, producer (brand), model, body types, (following) technical inspection date, set (features) of cars and so on.

## 2 Experimental design, materials, and methods

In Latvia, EU it is possible to observe market advertisements in internet with Data Mining technologies. There was collected average 21 thousands offers per months of used-car in Latvia, it is 3 times more offers then available in Latvian Vehicle Trade Register Information System [2], which is official state register, which was established on the basis of the LR Cabinet of Ministers Regulations No. 876 from 18 December 2007 [3]. This shows the advantage of the Data Science and Data Mining methods for collecting and providing information for different purposes.

The database was collected from biggest in the Latvia advertisement website www.ss.com [4] from its transport and cars section, by monthly repeated following R studio data scraping code:

library(Rcrawler)
Rcrawler(Website = "https://www.ss.com/lv/transport/cars/", KeywordsFilter = c("auto"), no_cores = 4, no_conn = 4, 6, ExtractXpathPat = c(
  "//*[@id=׳tdo_8׳]",
  "//*[@id=׳tdo_31׳]",
  "//*[@id=׳tdo_18׳]",
  "//*[@id=׳tdo_15׳]",
  "//*[@id=׳tdo_35׳]",
  "//*[@id=׳tdo_16׳]",
  "//*[@id=׳tdo_17׳]",
  "//*[@id=׳tdo_32׳]",
  "//*[@id=׳tdo_223׳]",
  "//b[@class=׳auto_c׳]"),
PatternsNames = c("Price","Model","First_registration_date","Motor", "Gearbox",
  "Mileage","Color","Body_types","Following_technical_inspection_date","Set_of_car"))
Price<-unlist(lapply(DATA, `[[`, 1))
Model<-unlist(lapply(DATA, `[[`, 2))
First_registration_date<-unlist(lapply(DATA, `[[`, 3))
Motor<-unlist(lapply(DATA, `[[`,4))
Gearbox<-unlist(lapply(DATA, `[[`,5))
Mileage<-unlist(lapply(DATA, `[[`,6))
Color<-unlist(lapply(DATA, `[[`,7))
Body_types<-unlist(lapply(DATA, `[[`,8))
Following_technical_inspection_date<-unlist(lapply(DATA, `[[`,9))
Set_of_car<-unlist(lapply(DATA, `[[`,10))
mytable<-data.frame(Price, Model, First_registration_date, Motor, Gearbox, Mileage, Color, Body_types, Following_technical_inspection_date, Set_of_car)
completeFun <- function(data, desiredCols) {
completeVec <- complete.cases(data[, desiredCols])
return(data[completeVec, ])}
write.csv(completeFun(mytable, "Price"), "auto.csv", row.names=F))

The data source represent biggest part of Latvian advertisements, the close competitor, reklama.lv [5], have has seven times less ads, so it is concluded, that the attached database fully represent Latvian used-car market in 2018. Collected data was compared with EU data [6].

In Riga Technical University monitoring process was started based on Latvian transport system leading experts [7], [8], [9] opinions about research topically for whole EU, Latvia and at regional level [10], [11], [12]. Parallel to data mining, RTU conduct different researches related both transport, logistic [13], international relations [14], petroleum products research [15] and several researches related with tax [16], [17], education [18] and business [19]. For researches use different economical – mathematical methods, as optimization method [20], econometric method [21], ratio analyse method [22], as well as methods and approach development [23], [24].

The relevance of the study is confirmed by the situation in the EU in November – December of 2018. In Belgium and France after diesel cars׳ tax increase had start riots [25]. In Germany, the situation is not so tense, given that for Germany there is the possibility of exporting diesel cars to the Eastern European countries.

## Figures and Tables

**Fig. S1:**
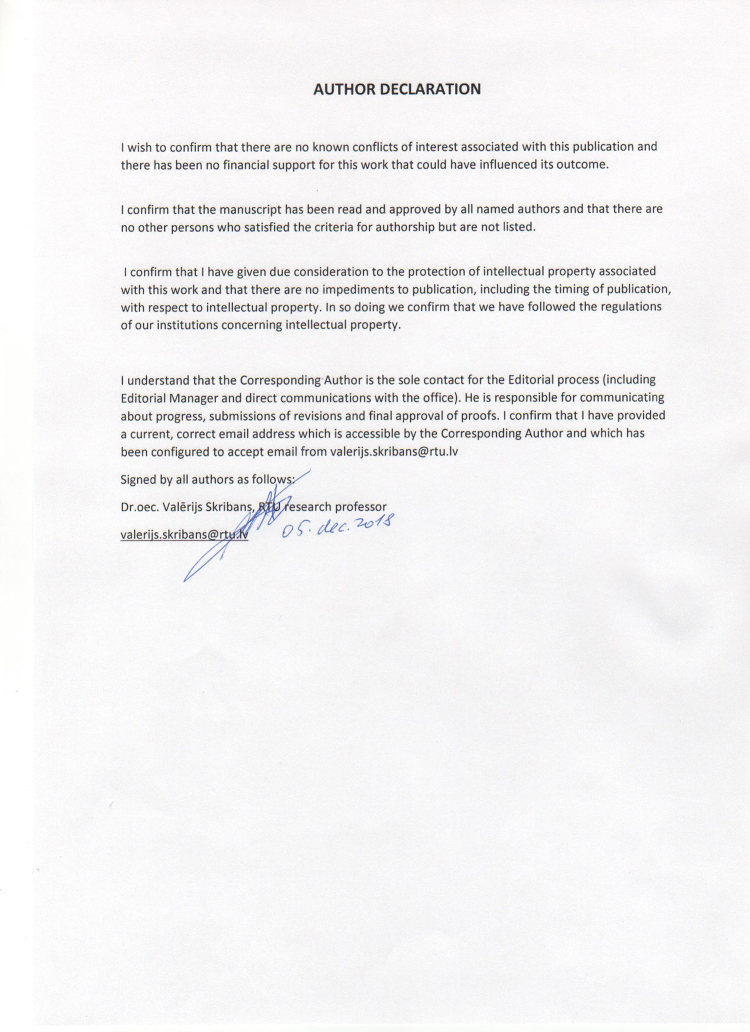
.

## References

[1] V. Skribans, S.W. Hegerty, Data Mining Application for Used-car Market Analysis, 2018. (In press).

[2] LVTRIS, Latvian Vehicle Trade Register Information System, 2018. 〈https://e.csdd.lv/tltr/〉 (Accessed 10 November 2018).

[3] LR MK, Ministru kabineta noteikumi Nr.876 Transportlīdzekļu un to numurēto agregātu tirdzniecības noteikumi, 2007. (Cabinet Ministry Regulation No.876 Regulations for the sale of vehicles and their numbered assemblies, in Latvian). 〈https://likumi.lv/doc.php?Id=168576〉 (Accessed 10 November 2018).

[4] Sludinājumi, Advertising Website, 2018. (in Latvian). 〈https://www.ss.com/lv/transport/cars/〉 (Accessed 10 November 2018).

[5] Reklāma, Advertising Website., 2018. (in Latvian). reklama.lv(Accessed 10 November 2018).

[6] ACEA, ACEA Report: Vehicles in Use Europe, 2017. 〈https://www.acea.be/uploads/statistic_documents/ACEA_Report_Vehicles_in_use-Europe_2017.pdf〉, 2018 (Accessed 10 November 2018).

[7] P. Patļins, Efficient transportation in cities and perishable goods secondary packaging, in: 15th International Scientific Conference "Engineering for Rural Development": Proceedings, vol. 15, Latvia, Jelgava, ISSN 1691–5976, 25–27 May 2016, pp. 1395–1401.

[8] Hudenko J., Ribakova N., Počs R. (2016). Cost that is directly incurred as a result of operating the train service on the 1520 mm rail with primarily freight transportation. Transp. Res. Procedia.

[9] P. Patļins, R. Počs, Secondary and tertiary packaging improvement opportunities into logistics processes, in: Proceedings of the 13th international Conference of Industrial Logistics, Poland, Zakopane, 209–2016, 28 Sep–1 Oct, 2016.

[10] Supe L., Jurgelāne I. (2017). Development trends in the national economy sectors in the Baltic States in 2005–2015. Res. Rural Dev. 2017.

[11] L. Jankova, T. Grizāne, I. Jurgelāne, Impact of the factors of the social capital of Zemgale Region on the development, in: Research for Rural Development: Annual 23nd International Scientific Conference Proceedings, Latvia, Jelgava, vol. 2, 17–19 May, 2017, pp. 256–263. (ISSN 1691-4031. e-ISSN 2255-923X).

[12] A. Auziņa-Emsiņa, V. Ozoliņa, High-technology industries competitiveness and regional allocation by nuts 3 regions in Latvian, in: Proceedings of the Research for Rural Development 2017: Annual 23rd International Scientific Conference: Proceedings, Latvia, Jelgava, vol. 2, 2017, 17-19 May 2017, pp. 241–248. (ISSN 1691–4031. e-ISSN 2255-923X). Available from: doi:〈 10.22616/rrd.23.2017.074〉.

[13] P. Patļins, R. Počs, Small shipment delivery’s quality improvement in cities with unstable traffic, in: Proceedings of the 30th European Conference on Modelling and Simulation, ECMS 2016: Proceedings, Germany, Regensburg, Regensburg: European Council for Modelling and Simulation, 31–3 May 2016, pp. 211–216. (ISBN 978-0-9932440-2-5).

[14] E. Kliedere, I. Jurgelāne, The role of digitized services to improve international activities of banks, in: Research for Rural Development 2016: Annual Proceedings of the 22nd International Scientific Conference Proceedings, Latvia, Jelgava, vol. 1, 18–20 May 2016, pp. 261–268. (ISSN 1691-4031. e-ISSN 2255-923X).

[15] Jurušs M., Seile E. (2017). Application of loss rates for petroleum products due to natural wastage in customs procedures. Procedia Eng..

[16] Pētersone M., Ketners K., Laurinavičius A. (2016). Improvement of customs and tax authorities׳ performance. Public Policy Adm..

[17] M. Pētersone, K. Ketners, Improvement of customs and tax administration ICT system performance, in: Proceedings of theNo: Annual 23 International Scientific Conference "Research for Rural Development 2017", LLU, Latvia, Jelgava, 17–19 May, 2017, pp. 263–269. (ISSN 2255-923X. e-ISSN 1691-4031). Available from: doi:〈 10.22616/rrd.23.2017.042〉.

[18] I. Jurgelāne, T. Grizāne, L. Jankova, Role of university lifelong learning process implementation, in: Research for Rural Development 2016: Annual Proceedings of the 22nd International Scientific Conference Proceedings, Latvia, Jelgava, vol. 2, 18–20 May 2016, pp. 246–254. (ISSN 1691-4031. e-ISSN 2255-923X).

[19] Grizāne T., Jurgelāne I. (2016). Social media impact on business evaluation. Procedia Comput. Sci..

[20] M. Zvirgzdiņa, O. Bogdanova, J. Spiridonovs, Aggregator as cost optimization tool for energy demand, in: Proceedings of the 17th International Scientific Conference "Engineering for Rural Development": Proceedings, University of Life Sciences and Technologies Faculty of Engineering, Latvia, Jelgava, vol. 17, 23–25 May, 2018, pp. 1781–1786. (ISSN 1691-5976). Available from: doi:〈 10.22616/ERDev2018.17.N322〉.

[21] Ozoliņa V., Auziņa-Emsiņa A. (2018). Modelling corporate income tax revenues in Latvia. Scientific Papers of the University of Pardubice. Ser. D: Fac. Econ. Adm..

[22] A. Auziņa-Emsiņa, V. Ozoliņa Evaluation of competitiveness of industries in regions and global market, in: Proceedings of the International Scientific Conference "Economic Science for Rural Development 2018", Latvia, Jelgava, 9–11 May, 2018, pp. 17–25. (e-ISBN 978-9984-48-293-4. ISSN 1691-3078. e-ISSN 2255-9930). Available from: doi:〈 10.22616/ESRD.2018.064〉.

[23] M. Krieviņa, V. Ozoliņa, Export target country selection tool for more competitive enterprises, in: Proceedings of the International Scientific Conference "Economic Science for Rural Development 2018", Latvia, Jelgava, 9–11 May, 2018, pp. 145–153. (e-ISBN 978-9984-48-293-4. ISSN 1691-3078. e-ISSN 2255-9930). Available from: doi:〈 10.22616/ESRD.2018.079〉.

[24] L. Kauškale, I. Geipele, Integrated approach of real estate market analysis in sustainable development context for decision making, Procedia Engineering, 2017. Vol. 172. Modern Building Materials, Structures and Techniques, MBMST, 2016. pp. 505–512. (ISSN 1877-7058). Available from: 〈 10.1016/j.proeng.2017.02.059〉.

[25] The Guardian, Paris riots: PM to Meet Protest Groups after Worst Unrest in Decade, 2018. 〈https://www.theguardian.com/world/2018/dec/02/paris-riots-worst-unrest-decade-with-shops-and-cars-set-alight-gilets-jaunes〉 (Accessed 4 December 2018).

